# FBP1-Altered Carbohydrate Metabolism Reduces Leukemic Viability through Activating P53 and Modulating the Mitochondrial Quality Control System In Vitro

**DOI:** 10.3390/ijms231911387

**Published:** 2022-09-27

**Authors:** Yi Xu, Lily Tran, Janet Tang, Vinh Nguyen, Elisabeth Sewell, Jeffrey Xiao, Christopher Hino, Samiksha Wasnik, Olivia L. Francis-Boyle, Ke K. Zhang, Linglin Xie, Jiang F. Zhong, David J. Baylink, Chien-Shing Chen, Mark E. Reeves, Huynh Cao

**Affiliations:** 1 Division of Hematology and Oncology, Department of Medicine, Loma Linda University, Loma Linda, CA 92354, USA; 2 Division of Regenerative Medicine, Department of Medicine, Loma Linda University, Loma Linda, CA 92354, USA; 3 Cancer Center, Loma Linda University, Loma Linda, CA 92354, USA; 4 Department of Pharmaceutical and Administrative Sciences, School of Pharmacy, Loma Linda University, Loma Linda, CA 92354, USA; 5 Department of Pathology & Human Anatomy, School of Medicine, Loma Linda University, Loma Linda, CA 92354, USA; 6 Department of Nutrition, Texas A&M University, College Station, TX 77030, USA; 7 Center for Epigenetics & Disease Prevention, Institute of Biosciences & Technology, College of Medicine, Texas A&M University, Houston, TX 77030, USA; 8 Department of Basic Science, School of Medicine, Loma Linda University, Loma Linda, CA 92354, USA

**Keywords:** AML, FBP1, P53, metabolism, glycolysis, OXPHOS, mitochondrion, autophagy, mitophagy

## Abstract

Acute myeloid leukemia (AML)—the most frequent form of adult blood cancer—is characterized by heterogeneous mechanisms and disease progression. Developing an effective therapeutic strategy that targets metabolic homeostasis and energy production in immature leukemic cells (blasts) is essential for overcoming relapse and improving the prognosis of AML patients with different subtypes. With respect to metabolic regulation, fructose-1,6-bisphosphatase 1 (FBP1) is a gluconeogenic enzyme that is vital to carbohydrate metabolism, since gluconeogenesis is the central pathway for the production of important metabolites and energy necessary to maintain normal cellular activities. Beyond its catalytic activity, FBP1 inhibits aerobic glycolysis—known as the “Warburg effect”—in cancer cells. Importantly, while downregulation of FBP1 is associated with carcinogenesis in major human organs, restoration of FBP1 in cancer cells promotes apoptosis and prevents disease progression in solid tumors. Recently, our large-scale sequencing analyses revealed FBP1 as a novel inducible therapeutic target among 17,757 vitamin-D-responsive genes in MV4-11 or MOLM-14 blasts in vitro, both of which were derived from AML patients with FLT3 mutations. To investigate FBP1′s anti-leukemic function in this study, we generated a new AML cell line through lentiviral overexpression of an *FBP1* transgene in vitro (named FBP1-MV4-11). Results showed that FBP1-MV4-11 blasts are more prone to apoptosis than MV4-11 blasts. Mechanistically, FBP1-MV4-11 blasts have significantly increased gene and protein expression of P53, as confirmed by the P53 promoter assay in vitro. However, enhanced cell death and reduced proliferation of FBP1-MV4-11 blasts could be reversed by supplementation with post-glycolytic metabolites in vitro. Additionally, FBP1-MV4-11 blasts were found to have impaired mitochondrial homeostasis through reduced cytochrome c oxidase subunit 2 (COX2 or MT-CO_2_) and upregulated PTEN-induced kinase (PINK1) expressions. In summary, this is the first in vitro evidence that FBP1-altered carbohydrate metabolism and FBP1-activated P53 can initiate leukemic death by activating mitochondrial reprogramming in AML blasts, supporting the clinical potential of FBP1-based therapies for AML-like cancers.

## 1. Introduction

Acute myeloid leukemia (AML) is a heterogeneous severe hematological malignancy [1,2]. Despite recent improvements in our understanding of AML and the active development of alternative treatments, about 50% of patients will relapse following induction chemotherapy [3], resulting in a dismal 5-year relative survival rate (30.5%, NCI SEER). Therefore, finding an effective treatment to overcome relapse and therapy resistance is an unmet need for many AML patients.

In a recent study, we performed RNA-sequencing (RNA-Seq) of four different AML-FLT3 (FMS-like tyrosine kinase 3) cell lines and discovered 17,757 genes responding to vitamin D treatment in vitro. In particular, fructose-bisphosphatase 1 (*FBP1*) stands out as the only gene with a 250-fold increase in expression [4]. *FBP1* is known to encode the gluconeogenic enzyme fructose-1,6-bisphosphatase, which catalyzes the hydrolysis of fructose 1,6-bisphosphate to fructose 6-phosphate and inorganic phosphate. The homologs of the human *FBP1* gene are conserved in 200 organisms, and RNA-Seq of 95 human individuals determined the tissue-specificity of *FBP1* gene expression to be prevalent only in major organs such as the liver with a reads per kilobase of transcript per million reads mapped (RPKM) of 175.9, kidneys (RPKM 118.4), lungs (RPKM 52.7), stomach (RPKM 31.9), and prostate (RPKM 10.4) [5]. Importantly, FBP1 has been found to suppress glycolysis and tumorigenic proliferation. On the other hand, FBP1 deficiency is associated with carcinogenesis and worsened clinical features in various solid tumors [6,7,8,9,10]. Moreover, restoration of FBP1 by overexpression of FBP1 in cancer cells was found to promote cell apoptosis and prevent disease progression in solid tumors [8,11,12,13]. However, the human *FBP1* gene has a lower expression in the bone marrow (RPKM 3.8) compared to that in solid organs [5]. The question of whether FBP1 plays a direct role in preventing leukemic progression and relapse remains to be determined. We recently discovered that vitamin D treatment in vitro significantly induced both FBP1 gene and protein expression intracellularly in various leukemic cell lines, including MV4-11, MOLM-14, and HL60 (a human acute promyelocytic leukemia (APL) cell line) [4]. Therefore, the therapeutic role of vitamin-D-induced FBP1 overexpression in leukemia remains a viable option that should be further explored.

In this study, we hypothesized that overexpressing *FBP1* in AML blasts would reduce leukemic viability by promoting blast apoptosis in vitro. By using the human AML cell line MV4-11, we generated a new AML FBP1-MV4-11 cell line with an overexpression of *FBP1* in vitro. We then analyzed the molecular phenotypes of FBP1-MV4-11 blasts by utilizing different experimental approaches, including flow cytometry (FC), Western blotting (WB), immunocytochemistry (ICC), and real-time polymerase chain reaction (qPCR). Here, we show that overexpression of *FBP1* reduced the concentration of pyruvate and promoted blast apoptosis by activating P53 in vitro. Additionally, our data suggested that FBP1-altered carbohydrate metabolism can affect the normal function of the mitochondria and interfere with metabolic homeostasis in AML blasts, establishing a potential foundation for the utilization of FBP1-based therapeutics to treat AML in vivo.

## 2. Materials and Methods

The list of reagents—including manufacturers and catalog numbers of antibodies, kits, and qPCR primers—can be found in the Supplementary Materials (Supplementary Tables S1 and S2). For this study, all experiments were repeated three times.

### 2.1. Cell Culture of MV4-11 and FBP1-MV4-11 Cell Lines

MV4-11 is a human-derived AML blast cell line with a FLT3 mutation (ATCC CRL-9591). The AML cell lines were cultured in RPMI-1640 medium (HyClone, Thermo Scientific, Waltham, MA, USA) supplemented with 10% heat-inactivated fetal bovine serum (FBS, HyClone, Citiva, Brooklin, NY, USA) and 100 U/mL penicillin/streptomycin. Cells were grown at 37 **°**C in a humidified atmosphere containing 5% CO_2_.

### 2.2. Preparation of FBP1 Lentivirus and Generation of FBP-MV4-11 Cell Lines In Vitro

**Figure 1:**
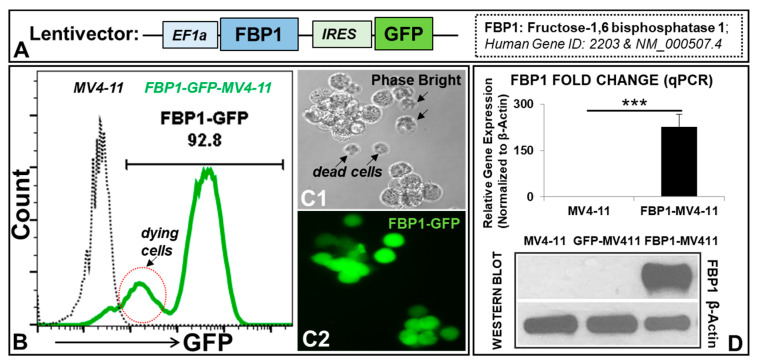
Generation of an AML cell line genetically bioengineered to overexpress *FBP1* (FBP1-MV411 blast) in vitro: (**A**) Schematic diagram of the lentiviral expression construct containing the human *FBP1* open reading frame (ORF) and GFP reporter, with the EF1a and IRES promoters, respectively. (**B**) A representative FC histogram showing GFP expression in FBP1-MV4-11 (FACS-sorted, see Materials and Methods) and MV4-11 cells; dying cells have weak GFP expression, indicated by a red circle. (**C1**) A representative phase-bright image (20×) of FBP1-MV4-11 cells; dying cells are indicated by arrows. (**C2**) A fluorescent image (20×) of the same FBP1-MV4-11 cells from (**C1**) showing GFP expression in viable cells, while dead cells no longer display fluorescence. (**D**) Overexpression of the FBP1 gene and protein in the FBP1-MV4-11 cell line, as confirmed by qPCR (fold change, upper panel) and Western blot assays (lower panel); the lentiviral empty construct has a GFP reporter without the *FBP1* ORF as the control (GFP-MV4-11). Where applicable, data are means ± SEM, and were analyzed by Student’s “*t*”-test; *** *p* < 0.005, n = 3.

The lentiviral transfer plasmid contains a full-length open reading frame (ORF) of human *FBP1* (GeneCopoeia catalog: EX-Z5880-Lv225, Rockville, MD, USA). This plasmid expresses *FBP1* and GFP, controlled by the EF1 and IRES promoters, respectively (Figure 1A). A GFP empty vector (GeneCopoeia catalog: EX-NEG-Lv225) was used as the vector control, which has an EF1a promoter and IRES-GFP.

Lentiviruses were prepared as previously described [14]. Briefly, HEK-293T cells were cultured in complete Dulbecco’s modified Eagle medium (DMEM, Gibco, Waltham, MA, USA) containing 10% FBS and 100 U/mL penicillin/streptomycin. When the cells were 70–80% confluent, the culture medium was replenished, and a transfection solution containing an envelope, packaging, and transfer plasmid (*FBP1*) was added dropwise to the cells. After the cells were cultured at 37 °C and in 5% CO_2_ for 48 h, the supernatants were collected, filtered through a 0.45 µm filter, and centrifuged at 4800× *g* at 4 °C for 24 h. The virus pellet was reconstituted in PBS containing 5% glycerol and titrated by a GFP-based flow cytometry method. The typical titer of virus was 10^8^–10^9^ transducing units/mL. The MV4-11 cells were transduced with FBP1 lentivirus at a multiplicity of infection (MOI) of 5. Twenty-four hours later, the virus was removed, and the culture medium was replenished. The cells were cultured for 24 h and examined for transduction efficiency via fluorescence microscopy and flow cytometry. When necessary, the above transduction procedure was repeated one more time to create a cell line with two rounds of transduction. FBP1-MV4-11 cells were purified by fluorescence-activated cell sorting (FACS; Research Core Facility, School of Medicine, University of California, Riverside). To generate FBP1-MV4-11 rescue cell lines, extra doses of 1 mM sodium pyruvate (Gibco 11360070) and/or 2 mM L-glutamine (Gibco 25030081) were added to the culture medium to treat blasts in vitro.

### 2.3. P53 Promoter Assay In Vitro

A lentiviral plasmid containing the promoter reporter clone with Gaussia luciferase reporter for human TP53 (NM_001126115; GeneCopoeia catalog: HPRM34629-LvPG04-50) was acquired. The preparation of the lentivirus of the P53 promoter reporter was performed similarly to that of the FBP1 lentivirus previously mentioned. The P53 promoter reporter was transduced into MV4-11 and FBP-MV4-11 cell lines to generate P53-MV4-11 and P53-FBP1-MV4-11 blasts in vitro. The detection of P53 activity in blasts and their secreted supernatants was performed using the Secrete-Pair™ Dual Luminescence Assay Kit (GeneCopoeia, Rockville, MD, USA). Images were acquired through a high-resolution CCD camera (Perkin Elmer IVIS Lumina III, Waltham, MA, USA).

### 2.4. Flow Cytometry (FC)

Cells were harvested and examined for the expression of cell-surface biomarkers and intracellular proteins by multichromatic flow cytometry, as previously described [15]. About 0.5~1 × 10^6^ cells were resuspended in 100 µL FC buffer (PBS containing 1% FBS and 0.05% sodium azide) and stained with various fluorescence-conjugated antibodies specific to the desired cell-surface proteins at 4 °C for 30 min. The surface-stained cells were then fixed and permeabilized using the appropriate reagents (BD Pharmingen Cytofix/Cytoperm buffer, San Diego, CA, USA) and further stained with appropriate fluorescence-conjugated antibodies specific to the desired intracellular proteins at 4 °C for 2 h in the permeabilizing buffer (BD Perm/Wash buffer). Concentrations of the antibodies were used as per the manufacturers’ recommendations (Supplementary Table S1). Finally, the stained cells were washed twice in permeabilizing buffer and twice in FC buffer before analysis on the BD FACSAria II (Franklin Lakes, NJ, USA). Data were analyzed using FlowJo software (Tree Star Inc., Ashland, OR, USA).

### 2.5. RNA Isolation and qPCR Analysis

AML blasts were collected for RNA isolation and qPCR analysis as previously described [16]. Total RNA was isolated using the RNeasy Mini Kit (Qiagen, Hilden, DE) according to the manufacturer’s instructions. First-strand cDNA was synthesized using the SuperScript III Reverse Transcriptase (Invitrogen, Waltham, MA, USA). The qPCR was performed and analyzed using an Applied Biosystems 7900HT Real-Time PCR machine (Waltham, MA, USA). Sequences of the primers used in this study are available in Supplementary Table S2. The PCR conditions were 10 min at 95 °C followed by 40 cycles of 10 s at 95 °C and 15 s at 60 °C. The relative expression level of a gene was determined using the ΔΔCt method and normalized to β-actin.

### 2.6. Western Blotting (WB) Analysis

AML cells were collected for WB as previously described [17]. Briefly, cells were homogenized in an ice-cold cell lysis buffer composed of 20 mM HEPES (pH 7.5), 10 mM KCl, 1.5 mM MgCl_2_, 1 mM ethylenediaminetetraacetic acid, 1 mM dithiothreitol, 1 mM phenylmethylsulfonyl fluoride, 2 µg/mL of aprotinin, and 10 µg/mL of leupeptin, followed by incubation on ice for 30 min. The homogenates were ultrasonicated and centrifuged at 20,000× *g* for 30 min at 4 °C. Samples with equal quantities of protein were loaded onto 10% SDS–polyacrylamide gel and separated by electrophoresis at 100 V for 1 hr. Protein bands on the gel were then transferred onto Immobilon-P membranes (Millipore Corporation, Burlington, MA, USA) and probed with primary antibodies (Supplementary Table S1), followed by incubation with Amersham horseradish-peroxidase-conjugated secondary antibodies (Cytiva, Marlborough, MA, USA). The exposed films were then developed within minutes, with results quantified using the Kodak electrophoresis documentation and analysis system and Kodak ID image analysis software (Eastman Kodak, Rochester, NY, USA).

### 2.7. Immunocytochemistry (ICC) and Imaging Acquisition

ICC staining of FBP1-MV4-11 cells was performed as reported previously [18]. Images were taken using an Olympus 1X71 fluorescent microscope and processed using the Olympus cellSens Dimension 1.15 imaging software (Tokyo, Japan).

### 2.8. Pyruvate Assay

The cell numbers of each experimental group were counted to ensure equal cell numbers per sample. The cell samples were then processed according to the manufacturer’s protocol (Sigma-Aldrich catalog: MAK332, St. Louis, MO, USA). Briefly, the lactate concentration of each sample was determined by an enzymatic assay, resulting in a colorimetric product proportional to the concentration of pyruvate present. The experimental plates were read on a spectrophotometric microplate reader at 570 nm. The pyruvate concentration within the samples was calculated by comparing the sample OD to the standard curve.

### 2.9. Statistical Analysis

Statistical analyses were performed with GraphPad software (Prism 5.02, San Diego, CA, USA). The quantitative analyses were analyzed with 1- or 2-way ANOVA, followed by Dunnett’s multiple comparisons test, Bonferroni’s post hoc analysis, or an unpaired *t*-test, as appropriate. All values were presented as the mean ± SEM. Results were considered statistically significant when the *p*-value was <0.05.

## 3. Results

### 3.1. Generation of an AML Cell Line Overexpressing FBP1 (FBP1-MV411 Blast)

To investigate novel anti-leukemic functional roles of FBP1 in leukemic cells, we overexpressed the *FBP1* gene in MV4-11 blasts via lentiviral transduction in vitro. FBP1 overexpression was assessed by GFP expression from the same lentiviral construct (Figure 1A) and confirmed with PCR and Western blots. The FC data demonstrated that about 92.8% of the MV4-11 blasts were successfully transduced, confirming the establishment of a viable FBP1-MV4-11 blast line (Figure 1B). Additionally, both phase-bright and fluorescent microscope imaging confirmed that viable leukemic cell clusters were GFP+ ^high^, dying cells were GFP+ ^low^ (indicated by red circles, Figure 1B), and dead cells were not fluorescent (indicated by arrows, Figure 1C). Finally, both qPCR and Western blotting assays confirmed the successful generation of the FBP1-MV4-11 cell line by showing significantly increased FBP1 transgene and protein expressions when compared to the native MV4-11 or GFP-MV4-11 control cell lines (Figure 1D).

### 3.2. Molecular Phenotypes of the FBP1-MV4-11 Cell Line In Vitro

**Figure 2:**
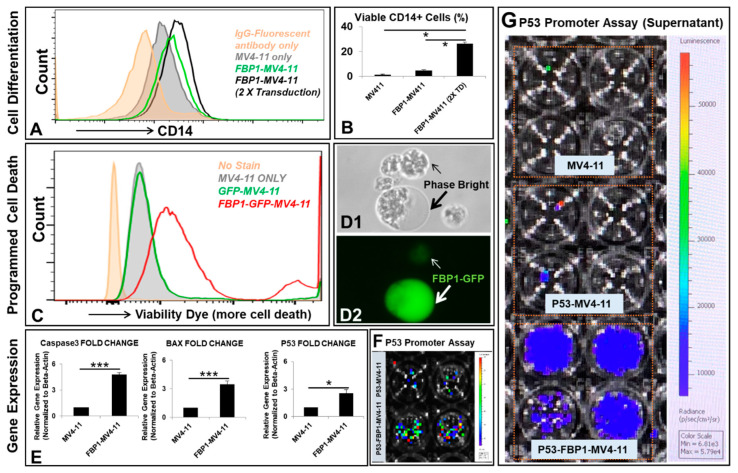
Molecular phenotypes of the FBP1-MV4-11 cell line in vitro: (**A**) A representative FC histogram showing CD14 expression in different MV4-11 cell lines, including naïve MV4-11, FBP1-MV4-11, and FBP1-MV4-11 cells with double FBP1 lentiviral transduction; the histogram of GFP+ expression in these cell lines can be found in Supplementary Figure S1. (**B**) Cumulative FC percentage data of CD14+ cells in different MV4-11 cell lines from (**A**). (**C**) A representative FC histogram showing the expression of viability dye (more expression representing more cell death) in FBP1-MV4-11 (red line plot), GFP-MV4-11 (green line plot), and MV4-11 cells (filled gray plot); the filled orange plot represents the unstained control. ***(*D1**,**D2**) Representative phase-bright and fluorescent images (20×) showing some GFP+ FBP1-MV4-11 cells that were abnormally larger (indicated by a large arrow) than their neighboring cells; the small arrow indicates a dying cell with weak GFP+. (**E**) Gene expression of programmed cell death proteins in FBP1-MV4-11 cells was analyzed by qPCR; data of mRNA expression show the fold change (normalized to β-actin) of genes encoding caspase-3, BAX, and P53. (**F,G**) P53 promoter assay of blasts and their supernatants was performed (see details in the Materials and Methods); representative live images show luciferase activity in MV4-11 and FBP1-MV4-11 blasts (**F**) and supernatants from different experimental groups (**G**). Where applicable, data are means ± SEM and were analyzed by Student’s “*t*”-test; * *p* < 0.05, *** *p* < 0.005, n = 3.

Next, we performed analyses of the functional properties of the FBP1-MV4-11 cell line in vitro. To examine FBP1′s function in blast differentiation, three cell lines were analyzed by flow cytometry, including MV4-11, an FBP1-MV4-11 cell line with one round of FBP1 lentiviral transduction, and an FBP1-MV4-11 cell line with two rounds of FBP1 lentiviral transduction (Figure 2A). The cell line with two rounds of lentiviral transduction (higher expression of *FBP1*, Supplementary Figure S1) demonstrated a significantly higher percentage (CD14+: 27.05%) of viable CD14+ blasts (a biomarker of monocytic differentiation [17]) when compared to both the naïve control (CD14+: 1.287%) and the cell line (CD14+: 5.14%) with only one round of FBP1 lentiviral transduction, indicating that increased *FBP1* overexpression could lead to enhanced differentiation of leukemia cells (Figure 2B).

Flow cytometry analysis was also conducted to observe the effects of *FBP1* gene expression on cell viability. The FC histogram demonstrated that increased FBP1 expression was directly correlated with increased death of FBP1-MV4-11 cells when compared to MV4-11 or GFP-MV4-11 blasts (Figure 2C). These FC findings are consistent with previous papers that hypothesized FBP1′s role in suppressing glycolysis and promoting apoptosis, leading to inhibition of tumorigenic proliferation [6,7,8,9,10]. Interestingly, the phase-bright analysis showed certain FBP1-MV4-11 cells with membrane blebbing that were abnormally larger than their neighboring blasts, which displayed cellular shrinkage and fragmentation (indicated by arrows, Figure 2D)—a finding associated with caspase-3-induced apoptosis [19].

To elucidate the mechanism of blast death, we conducted additional qPCR analysis on each cell line. The FBP1-MV4-11 cell line was found to have significantly increased gene expression of key pro-apoptotic mediators, including a 4.7-fold increase in caspase-3 and a 3.4-fold increase in Bcl-2-associated X protein (BAX, Figure 2E). In addition, FBP1 expression also significantly increased the gene expression of P53—a tumor-suppressor gene (2.5-fold increase, Figure 2E) known to initiate programmed cell death in leukemic cells [20]. Finally, to confirm whether FBP1 activates the promoter of the P53 gene, we lentivirally transduced a P53 promoter reporter construct into FBP1-MV4-11 and MV4-11 cells. Luciferase activity was measured in both blasts and secreted supernatants of each cell line via the dual-luciferase assay kit (GeneCopoeia) (Figure 2F, G). Increased luciferase radiance in the FBP1-MV4-11 cell line suggests a strong correlation between FBP1 overexpression and P53 induction. More specifically, luciferase analysis demonstrated FBP1′s role in activating the P53 gene promoter, supporting our hypothesis that increased *FBP1* gene expression leads to increased anti-tumorigenesis properties.

### 3.3. Pyruvate and/or Glutamine Reversed Cell Death and Reduced the Proliferation of FBP1-MV4-11 Cells by Inhibiting P53 Expression In Vitro

**Figure 3:**
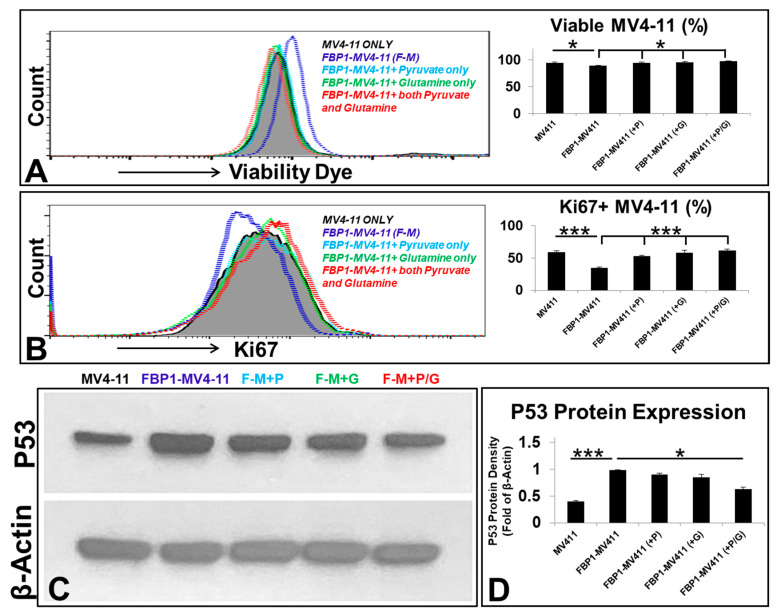
The reduced proliferation of FBP1-MV4-11 cells could be rescued by supplementation of pyruvate and/or glutamine in vitro: (**A**) A representative FC histogram showing the expression of viability dye (more expression representing more cell death) in different FBP1-MV4-11 rescue cell lines in vitro, including naïve MV4-11, FBP1-MV4-11 cells, FBP1-MV4-11 cells with supplementation of pyruvate, FBP1-MV4-11 cells with supplementation of glutamine, and FBP1-MV4-11 cells with supplementation of both pyruvate and glutamine; details of pyruvate and glutamine dosages and culture conditions are described in the Materials and Methods; *Right panel:* Cumulative FC percentage data of viable blasts in different experimental groups from (**A**). (**B**) A representative FC histogram showing Ki67 expression in different FBP1-MV4-11 rescue cell lines in vitro; *Right panel:* Cumulative FC percentage data of Ki67+ expression in different experimental groups from (**B**). (**C**) A representative film showing WB assay of human P53 protein expression in different experimental groups from (**A**). (**D**) Cumulative WB data of human P53 expression in different experimental groups from (**C**). Where applicable, data are means ± SEM and were analyzed by Student’s “*t*”-test; * *p* < 0.05, *** *p* < 0.005, n = 3.

Next, to investigate the possibility of reversing the inhibitory effect of FBP1 on blast proliferation, we rescued FBP1-MV4-11 cells by supplementing them with pyruvate and/or glutamine in vitro. We hypothesized that the supplementation of key metabolites would allow blasts to bypass the glycolytic pathway that was inhibited by FBP1 through alternate energy-producing pathways to facilitate their continued survival and proliferation. FBP1-MV4-11 blasts were found to have significantly increased cell death (viable cells: 88.3%) and decreased expression of Ki67 (34.7%)—a biomarker for the proliferation of AML blasts—when compared to MV4-11 (viable cells: 95.6% and Ki67+: 59.1%) in vitro (Figure 3A,B). Interestingly, the addition of pyruvate (viable cells: 94.1% and Ki67+: 53.3%), glutamine (viable cells: 95.2% and Ki67+: 58.4%), and pyruvate + glutamine (viable cells: 97.4% and Ki67+: 61.3%) increased the percentage of viable blasts and accelerated the proliferation of FBP1-MV4-11, as shown by the increased percentage of Ki67+ expression (Figure 3A,B). Finally, to investigate a mechanistic link between FBP1-activated P53 and phenotypes of leukemic progression, we compared the protein expression of P53 in each cell line. Both pyruvate and glutamine individually reduced P53 in FBP1-MV4-11 cells in vitro. However, the combination of pyruvate and glutamine had a much stronger inhibitory effect on P53 expression than each single agent (P53 protein density normalized to β-actin: 0.39 in MV4-11 versus 0.98 in FBP1-MV4-11, and 0.63 in FBP1-MV4-11 (+P/G); Figure 3C,D), showing an additive effect between glutamine and pyruvate in leukemic metabolism. These results also suggest that increased shunting of metabolism past the bottleneck point of our induced *FBP1* overexpression led to decreased cellular apoptosis and continued leukemic growth.

### 3.4. Mitochondrial Metabolic Reprogramming Responds to Disturbed Metabolic Homeostasis in the FBP1-MV411 Cell Line

**Figure 4:**
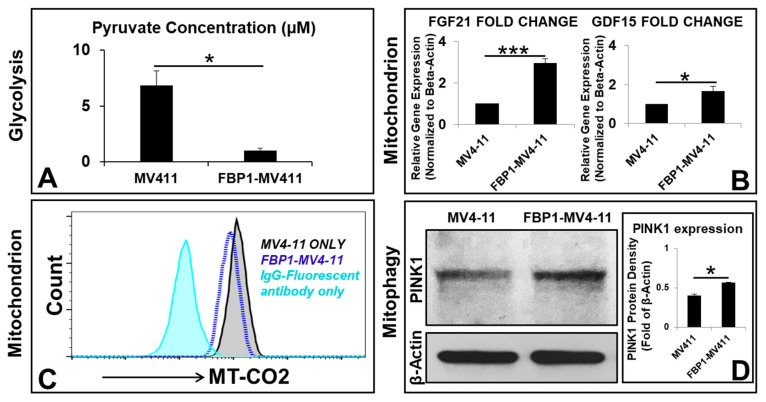
Mitochondrial adaptation responds to impaired metabolic homeostasis in the FBP1-MV411 cell line in vitro: (**A**) Intracellular concentrations of pyruvate in MV4-11 and FBP1-MV4-11 cells were measured by using the Pyruvate Assay Kit. (**B**) Gene expression of biomarkers for mitochondrial dysfunction in FBP1-MV4-11 cells was analyzed by qPCR; data of mRNA expression show the fold change (normalized to β-actin) of genes encoding FGF21 and GDF15. (**C**) A representative FC histogram showing the expression of COX2 (cytochrome c oxidase subunit 2, MT-CO2) in FBP1-MV4-11 (blue line plot) and MV4-11 cells (black line plot); the filled sky-blue plot represents the IgG fluorescent control. (**D**) A representative film showing WB assay of human PINK1 (PTEN-induced kinase) protein expression in MV4-11 and FBP1-MV4-11 cells; *Right panel*: Cumulative WB data of human PINK1 expression from (**C**). Where applicable, data are means ± SEM and were analyzed by Student’s “*t*”-test; * *p* < 0.05, *** *p* < 0.005, n = 3.

Next, we investigated the correlation between FBP1-altered carbohydrate metabolism and mitochondrial adaptive pathways. The mitochondria, the central hub of energy production to support leukemic survival and proliferation [21]—largely depend on metabolites generated from carbohydrate pathways, including major products of glucose such as pyruvate and NADH, whose deprivation induces leukemic apoptosis [22]. Pyruvate molecules produced by glycolysis are actively transported into the mitochondrial citric acid cycle (TCA or Krebs cycle) to generate ATP [23]. Therefore, we measured the intracellular concentration of pyruvate in FBP1-MV4-11 blasts. FBP1-MV4-11 blasts were found to have significantly decreased concentrations of pyruvate (0.99 µM) in comparison to those of MV4-11 blasts (6.83 µM) in vitro (Figure 4A). Although the TCA cycle utilizing oxidative phosphorylation (OXPHOS, aerobic respiration) can generate far more ATP (36 ATPs) than glycolysis (anaerobic respiration, 2 ATPs), the process depends on the availability of oxygen and metabolites in the leukemic microenvironment, emphasizing the vulnerability of biochemical processes in the mitochondria [24]. Therefore, these results suggest an inverse relationship between *FBP1* expression and glycolytic activity.

To elucidate the mechanism by which *FBP1* overexpression affects the mitochondria, we conducted additional qPCR studies. The FBP1-MV4-11 cell line was found to have significantly increased gene expression of fibroblast growth factor 21 (*FGF21*, 2.95-fold increase) and growth differentiation factor 15 (*GDF15*, 1.66-fold increase) (Figure 4B), which are biomarkers of mitochondrial dysfunction and OXPHOS deficiency [25,26]. Additionally, targeting electron transport chain (ETC) complex proteins has been found to shut down energy machinery and facilitate the leakage of pro-apoptotic proteins in the mitochondria [27]. Cytochrome c oxidase subunit 2 (COX2, or MT-CO2)—the functional core of cytochrome c oxidase—plays an essential role in the transfer of electrons in the OXPHOS process and, unfortunately, serves as an adverse prognostic marker for adult AML [28]. Interestingly, our results showed FBP1-MV4-11 blasts with significantly reduced MT-CO2, suggesting that FBP1 might also play a disruptive role in mitochondrial aerobic respiratory chains (Figure 4C).

In addition to building an abnormal energy production pathway to support uncontrolled blast proliferation, leukemic cells also utilize metabolic adaptation to create resistance to treatment [29]. Our protein analysis showed increased expression of PTEN-induced kinase (PINK1)—a biomarker of mitophagy [30]—indicating the activation of the mitochondrial quality control system in FBP1-MV4-11 blasts (PINK1 protein density normalized to β-actin: 0.4 in MV4-11 versus 0.56 in FBP1-MV4-11, Figure 4D). In summary, our findings suggest that FBP1-altered leukemic metabolism leads to the activation of mitochondrial adaptive pathways to maintain metabolic homeostasis, supporting the survival and continued proliferation of AML blasts.

## 4. Discussion

**Figure 5:**
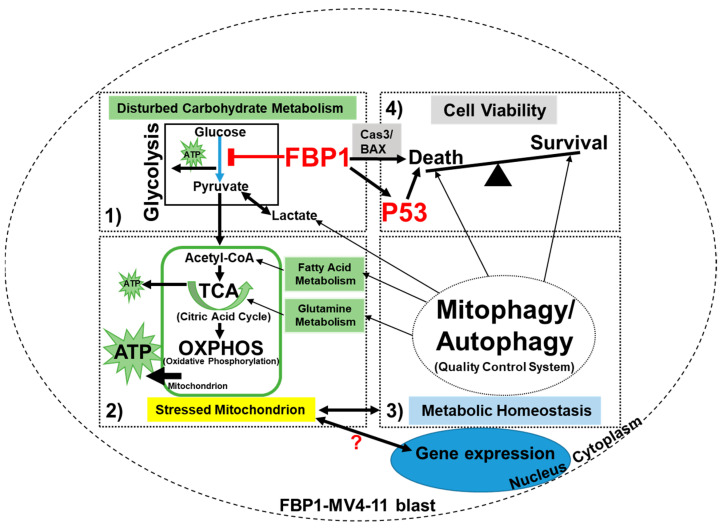
A schematic diagram of the anti-leukemic functional roles of FBP1 and FBP-activated P53 in AML blasts in vitro: There are multifaceted anti-leukemic functions of FBP1 in the treatment of AML blasts; FBP1 inhibits the process of glycolysis by reducing key intermediate metabolites such as pyruvate (**BOX1**) and regulates blast differentiation via changes in cellular signaling. FBP1-disturbed glycolysis can induce stress in the mitochondria (**BOX2**), leading to the reduction in COX2 in the OXPHOS process. To maintain metabolic homeostasis and survive, FBP1-MV4-11 blasts can activate the quality control system of mitochondria via upregulation of PINK1 (**BOX3**). The activated mitophagy and autophagy can replenish key metabolites such as fatty acids and glutamine, to either promote the regeneration of dysfunctional mitochondria (**BOX3**) or prevent cell death (**BOX4**). In addition to its glycolytic regulatory function, FBP1 can increase pro-apoptotic proteins and tumor suppressors—such as caspase-3, BAX, and P53—while decreasing hypoxic oncogenes such as HIF1A (not shown), leading to programmed leukemic cell death (**BOX4**).

In this study, we generated a new AML cell line that overexpresses the *FBP1* gene, and we found that FBP1 has a multifaceted impact on the regulation of leukemic survival and growth in vitro (Figure 5). Our data revealed that FBP1 simultaneously stimulated the apoptosis of blasts while also preventing leukemic growth through the regulation of pro-apoptotic and tumor-suppressive genes, such as caspase-3, BAX, and P53. Furthermore, the P53 promoter assay results suggest that one of the most significant non-catalytic regulatory functions of FBP1 is to stimulate the expression of the tumor-suppressor P53 gene, whose downregulation or mutation characterizes a distinct feature of AML [31,32]. Interestingly, increased P53 production in FBP1-MV4-11 blasts could be reversed by adding post-glycolytic metabolites, such as pyruvate and glutamine. Mechanistically, it was found that pyruvate-deprivation-induced cell-cycle arrest is associated with augmented P53 pathways via the upregulation of the P21 protein—a cyclin-dependent kinase inhibitor and target of P53 [33]. Moreover, P53 has been found to be regulated by aerobic glycolysis in cancer cells [34]. A similar relationship was also seen between glutamine and P53 [35]. Our data suggest that pyruvate and glutamine might provide the blasts with alternative energy-producing pathways to facilitate continued leukemic survival and proliferation through the decrease in P53 gene expression.

Another significant phenotype in FBP1-MV4-11 blasts is the activation of mitochondrial reprogramming. Previously, pyruvate deprivation was found to induce an accumulation of injured mitochondria [33]. Damaged mitochondria are targeted for degradation by an autophagy pathway known as mitophagy [36]. Interestingly, this pyruvate-mediated mitophagy was not affected by the OXPHOS or cellular ATP levels and is thus independent of energy metabolism [37], reflecting the complex nature of metabolic homeostasis and the mysterious crosstalk between the mitochondria and the nuclear transcription machinery [38]. The FBP1-mediated reduction in pyruvate and COX2 (MT-CO2) shown by our data demonstrates the tightly coupled relationship between glycolysis and OXPHOS, because pyruvate—the end product of glycolysis—is fuel for OXPHOS [39]. Therefore, FBP1-subverted glycolysis can be linked to an impaired OXPHOS process, leading to mitochondrial stress or dysfunction (BOX2, Figure 5). To maintain metabolic homeostasis and survival, FBP1-MV4-11 blasts can activate the mitochondrial repair process of mitophagy via upregulation of PINK1 (BOX3, Figure 5). Activated mitophagy and autophagy replenish key metabolites such as fatty acids and glutamine, to either promote the regeneration of dysfunctional mitochondria or prevent cell death (BOX4, Figure 5).

In summary, our current report provides the first evidence that FBP1 is associated with several non-catalytic pathways—including (1) inhibition of glycolysis, (2) activation of P53, and (3) promotion of blast differentiation—that ultimately lead to the reduction in leukemic cells. These results need to be further confirmed in other patient-derived cell lines or primary samples to broaden the relevance of this study, with the best scenario involving the investigation of the metabolic changes in murine leukemia models. Therefore, treatments inducing increased FBP1 production hold promise as a therapeutic strategy for AML patients and need to be investigated further.

## Data Availability

The datasets used and analyzed in the current study are available from the corresponding author.

## Supplementary Materials

The following supporting information can be downloaded at: https://www.mdpi.com/article/10.3390/ijms231911387/s1. Table S1: List of Reagents used in this study; Table S2: List of Primers (Origene, etc.) used in this study; Figure S1. GFP expression in different MV4-11 cell lines in vitro.
