# Supplementary material for: FBP1-Altered Carbohydrate Metabolism Reduces Leukemic Viability through Activating P53 and Modulating the Mitochondrial Quality Control System In Vitro
Source: Int J Mol Sci. 2022 Sep 27;23(19):11387. doi: 10.3390/ijms231911387 (PMC9570078; doi:10.3390/ijms231911387)
Supplement: Supplementary file 1 [file ijms-23-11387-s001.zip › ijms-1881145-supplementary.pdf]

FBP1-Altered Carbohydrate Metabolism Reduces Leukemic Viability through Activating P53 and Modulating the Mitochondrial Quality Control System In Vitro

Yi Xu[1, 2, 3@], Lily Tran[2], Janet Tang[2], Vinh Nguyen[2], Elisabeth Sewell[2], Jeffrey Xiao[2], Christopher Hino[1], Samiksha Wasnik[2], Olivia L. Francis-Boyle[4,5], Ke K. Zhang[6, 7], Linglin Xie[6], Jiang F Zhong[3, 8], David J. Baylink[2], Chien-Shing Chen[1, 3], Mark E. Reeves[1, 3], Huynh Cao[1,3]

Supplementary Documents

**List of Reagents**				
**Antibody/Reagents**	**Color**	**Cat. #**	**Company**	**Species Reactivity**
**Viability Dye eFluor™ 780**		65-0865-14	eBioscience	
**Ki67**	PE	350504	Biolegend	Human
**CD14**	APC	301808	Biolegend	Human
**CD44**	PE/CY7	338816	Biolegend	Human
**FBP1**		PIMA536138	Invitrogen	Human
**B-actin**		SC47778	Santa Cruz Biotechnology	Human
**MT-CO2 (COX2, N-20)**		SC-23983	Santa Cruz Biotechnology	Human
**Anti-goat IgG**	PE	F0107	R&D systems	
**P53**		645805	Biolegend	Human
**P53**		2524T	Cell Signaling Tech	Human
**PINK1**		846201	Biolegend	Human
**Pyruvate Assay Kit**		MAK332-1KT	Sigma Aldrich	
**Secrete-Pair™ Dual Luminescence Assay Kit**		LF031	GeneCopeia	

Supplementary Table S1: List of Reagents used in this study

#	**Name (HUMAN)**	**Forward Sequence**	**Reverse Sequence**
1	**FBP1**	GCAGTCAAAGCCATCTCTTCGG	TAACCAGGTCGTTGGAGAGGAC
2	**Caspase 3**	GGAAGCGAATCAATGGACTCTGG	GCATCGACATCTGTACCAGACC
3	**BAX**	TCAGGATGCGTCCACCAAGAAG	TGTGTCCACGGCGGCAATCATC

4	**P53**	CCTCAGCATCTTATCCGAGTGG	TGGATGGTGGTACAGTCAGAGC
5	**FGF21**	CTGCAGCTGAAAGCCTTGAAGC	GTATCCGTCCTCAAGAAGCAGC
6	**GDF15**	CAACCAGAGCTGGGAAGATTCG	CCCGAGAGATACGCAGGTGCA

Supplementary Table S2: List of Primers (Origene, etc.) used in this study

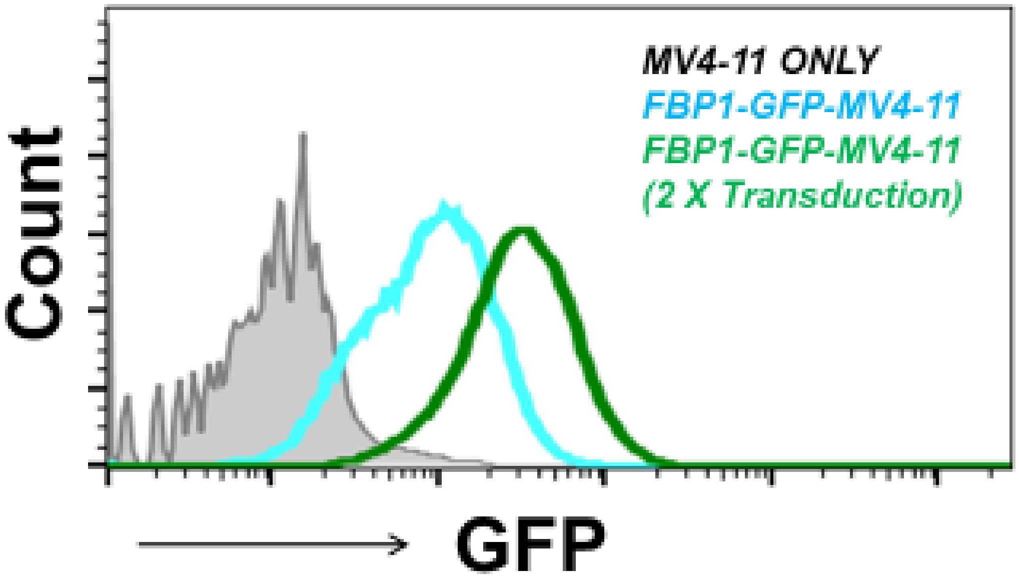

Supplementary Figure S1. GFP expression in different MV4-11 cell lines *in vitro*.

A representative FC histogram shows GFP expression in naïve MV4-11, FBP1-MV4-11 with one round of FBP1-lentiviral transduction and FBP1-MV4-11 cells with two rounds of FBP1-lentiviral tranduction.
